# MACULAR BLOOD FLOW CHANGES IN BRANCH RETINAL VEIN OCCLUSION EXAMINED BY OPTICAL COHERENCE TOMOGRAPHY ANGIOGRAPHY VARIABLE INTERSCAN TIME ANALYSIS

**DOI:** 10.1097/IAE.0000000000003596

**Published:** 2022-08-15

**Authors:** Naomi Nishigori, Yuki Muraoka, Masaharu Ishikura, Shin Kadomoto, Yuki Mori, Shogo Numa, Tomoaki Murakami, Sotaro Ooto, Akitaka Tsujikawa

**Affiliations:** Department of Ophthalmology and Visual Sciences, Kyoto University Graduate School of Medicine, Kyoto, Japan

**Keywords:** branch retinal vein occlusion, interscan time, macular edema, optical coherence tomography, variable interscan time analysis

## Abstract

To examine the relationship between changes in retinal blood flow and the recurrence of macular edema in eyes with branch retinal vein occlusion.

**Methods:**

This observational study included 32 eyes in 32 patients (18 men and 14 women) with branch retinal vein occlusion who visited the Department of Ophthalmology at Kyoto University Hospital (February 2021–November 2021). At the time of inclusion in the study, each patient underwent optical coherence tomography angiography on a macular area measuring 4 × 4 mm^2^. For variable interscan time analysis, different interscan times were set at 7.6 (IST_7.6_) and 20.6 ms (IST_20.6_) for the optical coherence tomography angiography. The parafoveal vessel densities were measured sectorally at IST_7.6_ and IST_20.6_, and their relationship with the longitudinal changes evident in the retinal thicknesses during the variable interscan time analysis examination and 2 months later was evaluated.

**Results:**

The parafoveal vessel densities in the affected sector was significantly greater at IST_20.6_ than at IST_7.6_ (*P* = 0.011). At 2 months after the variable interscan time analysis examination, 6 patients (19%) showed recurrence of macular edema involving the fovea. The difference in the parafoveal vessel densities (IST_20.6_ − IST_7.6_) in the affected sector was significantly associated with longitudinal retinal thickening in the corresponding parafovea (*P* = 0.020) and fovea (*P* = 0.014).

**Conclusion:**

In eyes with branch retinal vein occlusion, optical coherence tomography angiography variable interscan time analysis facilitated the detection of retinal blood flow changes that might be predictive for the recurrence of macular edema.

## Abstract 2

Supplemental Digital Content is Available in the Text.

Performing variable interscan time analysis in optical coherence tomography angiography on eyes with branch retinal vein occlusion enabled the detection of macular perfusion changes that were undetectable using the standard interscan times and might be predictive of the recurrence of macular edema.

Retinal vein occlusion (RVO) is one of the most common retinal vascular diseases.^1–3^ Retinal vein occlusion is often accompanied by macular edema (ME) and concomitant visual disturbances.^1^ The ME associated with RVO can be ameliorated using antivascular endothelial growth factor (VEGF) treatments.^4–7^ However, blockade of VEGF does not act on thrombus formation in the affected retinal veins, and the ME can recur if an intravitreal level of the anti-VEGF agent is decreased. It is also clinically difficult to predict the recurrence of ME.^8^

Until recently, the evaluation of retinal circulation was commonly performed using fluorescein angiography (FA). However, it is now more common to use optical coherence tomography (OCT) angiography (OCTA) in clinical research and practice. Optical coherence tomography angiography visualizes retinal blood flow by extracting decorrelation signals that change among multiple OCT-B scans acquired at the same location; this facilitates detection of retinal nonperfusion, microaneurysms, and collateral vessels in eyes with RVO.^9–13^ By contrast, it has also been reported that OCTA does not detect all of the lesions delineated by FA ^14^; this may be related to the association between the blood flow velocity and interscan time (IST) of OCTA.^15–17^ If the blood flow velocity is below the detection threshold of the IST, it theoretically cannot be visualized by OCTA.

In OCTA, a method to detect abnormal blood flow was recently developed; it uses changing ISTs and is called variable IST analysis (VISTA).^18^ Using VISTA, novel findings in diabetic retinopathy^19^ and age-related macular degeneration ^20,21^ were recently reported. If the VISTA technique can be applied to eyes with RVO, it might be possible to identify novel findings that are undetectable by conventional OCTA. However, researchers have not applied VISTA for eyes with RVO. Therefore, in this study, we used our VISTA protocol to study patients with branch RVO (BRVO) by examining their macular perfusion status and analyzing the changes in their retinal morphology.

## Methods

### Patients and Methods

This observational study was approved by the Institutional Review Board of Kyoto University Graduate School of Medicine (Kyoto, Japan), and it adhered to the tenets of the Declaration of Helsinki. Written informed consent was obtained from each subject before any study procedures or examinations.

We included patients with BRVO lasting more than six months in duration who visited the Department of Ophthalmology, Kyoto University Hospital, between February 2021 and November 2021. We excluded eyes with multiple retinal vein occlusions, BRVO located more to the nasal side than the optic disk, central RVO, hemicentral RVO, coexisting ocular disease (diabetic retinopathy, hypertensive retinopathy, retinal arterial occlusion, retinal arterial macroaneurysm, glaucoma, retinitis pigmentosa, and age-related macular degeneration), keratoconus, high myopia (more severe than −6 diopters), and/or high astigmatism (more severe than ±3 diopters). We also excluded eyes with poor-quality OCTA images (signal strength index of <6) caused by eye movement or media opacities.

At the initial visit, all the patients were treatment-naive and showed visual disturbance because of retinal hemorrhage and edema involving the fovea. The symptom duration was less than 3 months before the initial visit. The patients initially received intravitreal injections of ranibizumab (Lucentis, 0.5 mg/0.05 mL; Novartis Pharma AG; Basel, Switzerland) for the treatment of ME and/or serous retinal detachment at the fovea. None of the patients received treatment other than ranibizumab (e.g., bevacizumab or aflibercept injection, grid laser photocoagulation, steroid treatment, and surgical intervention). After the initial injections, pro re nata injections were performed only when the ME or serous retinal detachment was evident at the fovea on OCT images and the patient's informed consent could be obtained. For each patient, the same anti-VEGF agent was used for the initial and subsequent injections.

Eventually, 32 eyes of 32 consecutive patients (18 men and 14 women) with BRVO met the criteria and were included in the study. At the inclusion, the ME and retinal hemorrhage were substantially absorbed within the fields of OCTA views. Even if ME was present, its severity was insufficient to affect image quality.

### Variable Interscan Time Analysis in Optical Coherence Tomography Angiography

At the time of inclusion in the study, each patient underwent high-resolution spectral domain OCTA (OCT-A1, Canon Inc., Tokyo, Japan) in addition to routine examinations that included measurement of the best-corrected visual acuity (BCVA) using a Landolt chart, measurement of the intraocular pressure, slit-lamp biomicroscopy, and OCT.

For OCTA images, a foveal area (4 × 4 mm^2^; 464 × 464 pixels) was imaged in the rescan mode, where the scanner once captures the area from top to bottom and then goes back to rescan only the area that could not to be captured in the first scan.

For OCTA VISTA, a macular area was first scanned using an IST of 7.6 ms (IST_7.6_) and then scanned using an IST of 20.6 ms (IST_20.6_) in a repeat mode. This mode enables identical areas to be captured when the ISTs are changed. The patients were imaged three times at each IST, and averaged OCTA images were generated.

### Quantitative Measurements in Optical Coherence Tomography Angiography Variable Interscan Time Analysis

**Fig. 1.**
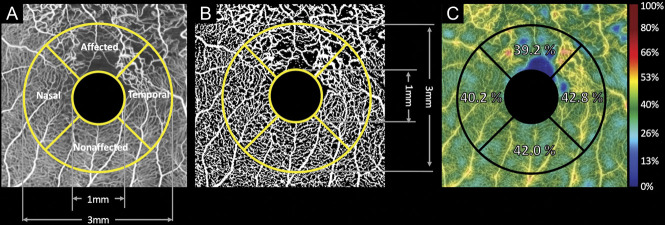
Quantitative sectoral evaluations of the macular retinal vasculature using OCTA for patients with branch retinal vein occlusion. **A.** Averaged OCTA images of the macular area, with a scanning area of 4 × 4 mm^2^. **B.** Binarized OCTA image. On the binarized image, the parafoveal VD is calculated as the ratio of the area occupied by the vessels to the total area of each sector. **C.** The VD color map made by the device's software program. These procedures (**A–C**) were performed using OCTA images acquired at ISTs of 7.6 milliseconds and 20.6 milliseconds for each patient.

For measuring parafoveal vessel densities (VD), the device's built-in software program was used to binarize the averaged OCTA images for the entire retina slab. The center of an Early Treatment Diabetic Retinopathy Study grid was set on the center of the fovea without any rotation (Figure 1). For each subfield in the inner (1–3 mm) ring of the Early Treatment Diabetic Retinopathy Study grid, the parafoveal VD was calculated as the ratio of the area occupied by the vessels to the total area (Figure 1). A color map of VD was created using the device's built-in software program (Figure 1). These procedures were performed using the OCTA images acquired at IST_7.6_ and IST_20.6_.

### Longitudinal Thickness Changes in the Fovea and Parafovea

At the time of the VISTA examination and 2 months afterward, we acquired macular volume scans using spectral domain OCT (Spectralis HRA + OCT, Heidelberg Engineering; Heidelberg, Germany) and created whole-retinal thickness maps centered on the fovea. In the Early Treatment Diabetic Retinopathy Study grid, the foveal thickness was defined as the averaged retinal thickness in the central grid; the parafoveal thickness was defined as the averaged retinal thickness in each sector of the inner ring. Retinal thickness calculations for each grid were performed using the manufacturer's built-in software program (Spectralis Acquisition and Viewing Modules, version 6.0; Heidelberg Engineering). The changes in the longitudinal thickness of the parafovea and fovea were evaluated, and their relationship with the differences in the parafoveal VDs between IST_20.6_ and IST_7.6_ were examined.

### Statistical Analysis

Statistical analyses were performed using JMP 16 (SAS Institute Inc., Cary, NC, USA). All values are presented as the mean ± SD. The parafoveal VDs acquired at IST_7.6_ and IST_20.6_ were compared for each sector using the paired *t*-test. The sectorial differences between the parafoveal VDs (IST_20.6_ − IST_7.6_) were compared using one-way analysis of variance and Tukey HSD test. A multivariate regression analysis was performed to evaluate the contribution of the parafoveal VDs differences (IST_20.6_ − IST_7.6_) to the changes in foveal thickness. Statistical significance was set at *P* < 0.05.

## Results

At the initial visit, all patients showed visual disturbance because of ME and/or serous retinal detachment (Table 1); the mean logarithm of the minimal angle of resolution BCVA was 0.17 ± 0.25 (Snellen equivalent: 20/200–20/13), and the mean foveal thickness was 471.8 ± 126.1 *µ*m. At the OCTA VISTA examination, the mean logarithm of the minimal angle of resolution BCVA improved to −0.05 ± 0.13 (Snellen equivalent: 20/40–20/12), and the foveal thickness decreased to 278.9 ± 57.5 *µ*m; both of these had improved significantly since the initial visit (*P* < 0.001 for each, Table 1).

**Table 1.** Characteristics of Included Patients With Branch Retinal Vein Occlusion

No. of patients included (men/women)	18/14
Age, years	68.3 ± 12.8
Systemic hypertension, (n)	18
Diabetes mellitus, (n)	5
Hyperlipidemia, (n)	10
At initial visit	
Mean logMAR visual acuity (range in Snellen equivalent)	0.17 ± 0.25 20/200–20/12
Mean foveal thickness, *μ*m	471.8 ± 126.1
Time from onset to OCTA examination, months	47.6 ± 37.2
Mean logMAR visual acuity (range in Snellen equivalent)	−0.05 ± 0.13 20/40–20/12
Mean foveal thickness, *μ*m	278.9 ± 57.5
No. of ranibizumab injections required	4.2 ± 4.1

Data are mean ± SD unless otherwise indicated.

### Parafoveal Vessel Densities Examined by Optical Coherence Tomography Angiography Variable Interscan Time Analysis

**Fig. 2.**
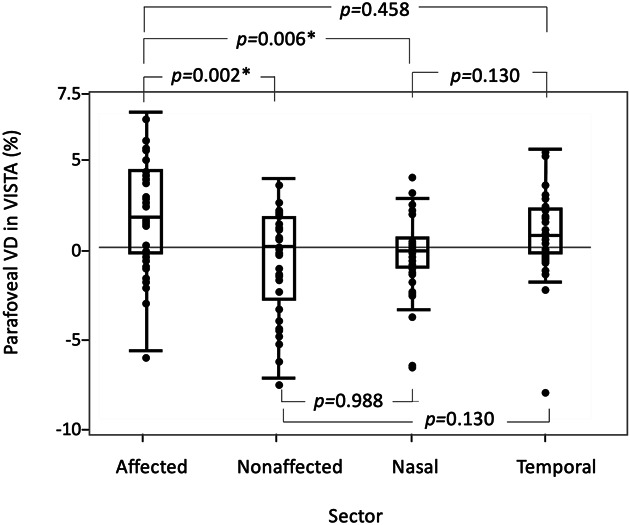
Comparisons of sector differences in the parafoveal VD examined by VISTA. In the VISTA examination, the difference in the parafoveal VD (calculated as the difference between the value measured using an interscan time of 20.6 milliseconds minus that of an interscan time of 7.6 milliseconds) in the affected sector was significantly greater than those in the unaffected and nasal sectors. *Significant difference.

**Fig. 3.**
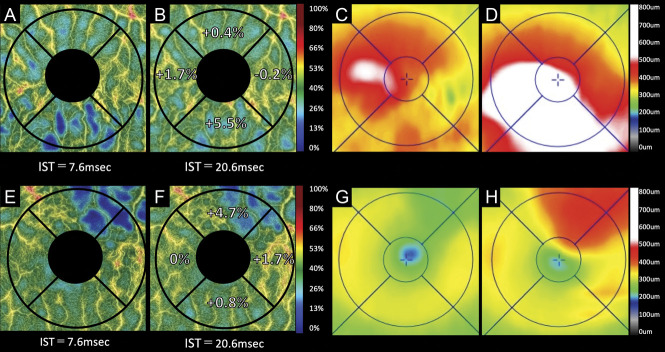
Two representative cases with recurrence of BRVO-associated macular edema examined by VISTA. **A**–**D.** A 73-year-old woman with inferotemporal BRVO in the left eye. Optical coherence tomography angiography acquired using IST of 7.6 milliseconds (**A**) and 20.6 milliseconds (**B**). **C** and **D.** Optical coherence tomography retinal thickness maps. At the initial visit, the Snellen visual acuity was 20/32. The patient received four anti-VEGF treatments for the macular edema before the VISTA examination. At the VISTA examination (**A**–**C**), the macular edema is substantially resolved, and the Snellen visual acuity improved to 20/12. The comparison between the OCTA images taken using ISTs of 20.6 milliseconds (**B**) and 7.6 milliseconds (**A**) shows that the parafoveal VDs increased in the affected and nasal sectors. Two months after the therapy (**D**), the macular edema recurred in the inferonasal parafovea where the parafoveal VD increased in the VISTA examination. **E**–**H.** A 70-year-old man with superotemporal BRVO in the left eye. Optical coherence tomography angiograms obtained using an IST of 7.6 milliseconds (**E**) and 20.6 milliseconds (**F**). **G** and **H.** Optical coherence tomography retinal thickness maps. At the initial visit, the Snellen visual acuity was 20/50. The patient received six anti-VEGF treatments for the macular edema before the VISTA examination. At the VISTA examination (**E**–**G**), the macular edema was substantially resolved, and the Snellen visual acuity improved to 20/12. A comparison between the images taken using ISTs of 20.6 milliseconds (**F**) and 7.6 milliseconds (**E**) shows that the parafoveal VD increased in the affected sector. Two months after the therapy (**H**), the macular edema recurred in the upper parafovea where the parafoveal VD increased in the VISTA examination.

**Supplemental Digital Content 1** (see **Table**, http://links.lww.com/IAE/B762) shows the mean parafoveal VDs for IST_7.6_ and IST_20.6_. In the affected sector, the mean parafoveal VD at IST_20.6_ was significantly greater than that at IST_7.6_ (*P* = 0.011). The mean difference in the parafoveal VD in the affected sector was significantly greater than that in the unaffected (*P* = 0.002) and nasal sectors (*P* = 0.006) (Figure 2). In 14 patients (43%), the parafoveal capillaries including collateral vessels and microaneurysms were lowly reflective, and the delineations were poor on the OCTA images at IST_7.6_; these were more reflective and more clearly delineated at IST_20.6_ (Figure 3; see **Figure**, **Supplemental Digital Content 2**, http://links.lww.com/IAE/B763) showing a BRVO-associated microaneurysm whose delineation is altered in VISTA.

### Association Between Variable Interscan Time Analysis Findings and Future Retinal Thickening

**Fig. 4.**
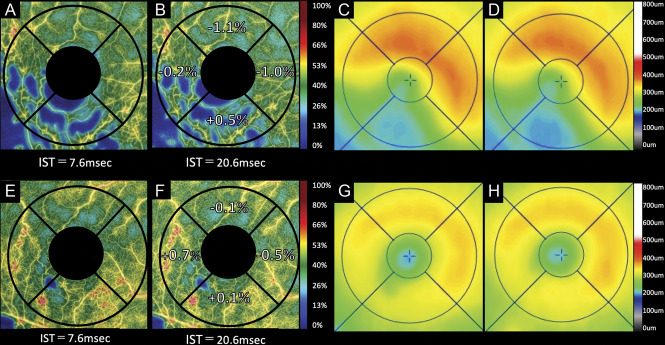
Two representative cases without recurrence of BRVO-associated macular edema examined by variable interscan time analysis (VISTA). **A**–**D.** A 57-year-old woman with inferotemporal BRVO in the right eye. Optical coherence tomography angiograms obtained using an IST of 7.6 milliseconds (**A**) or 20.6 milliseconds (**B**). **C** and **D.** Optical coherence tomography retinal thickness maps. At the initial visit, the Snellen visual acuity was 20/200. The patient received four anti-VEGF treatments for the macular edema before the VISTA examination. At the VISTA examination (**A**–**C**), the macular edema is substantially resolved, and the Snellen visual acuity improved to 20/15. A comparison between images taken using ISTs of 20.6 milliseconds (**B**) and 7.6 milliseconds (**A**) shows that the parafoveal VDs do not increase in the any parafoveal sectors. Two months after the VISTA examination (**D**), the macular edema had not recurred. **E**–**H.** A 63-year-old man with inferotemporal BRVO in the right eye. Optical coherence tomography angiograms obtained using ISTs of 7.6 milliseconds (**E**) and 20.6 milliseconds (**F**). **G** and **H.** Optical coherence tomography retinal thickness maps. At the initial visit, the Snellen visual acuity was 20/200. The patient received three anti-VEGF treatments for the macular edema before the VISTA examination. At the VISTA examination (**E**–**G**), the macular edema is substantially resolved, and the Snellen visual acuity improved to 20/12. The comparison between images obtained using ISTs of 20.6 milliseconds (**F**) and 7.6 milliseconds (**E**) shows that the parafoveal VD did not increase in the any parafoveal sectors. Two months after the VISTA examination (**H**), the macular edema had not recurred.

At 2 months after the VISTA examination, 6 patients (19%) exhibited recurrence of the ME involving the fovea. The differences in the parafoveal VDs (IST_20.6_ − IST_7.6_) of the affected and nasal sectors were significantly associated with parafoveal thickening in the corresponding sectors (*P* = 0.020 and *P* = 0.011, respectively; Table 2, Figures 3 and 4). In addition, the difference between IST_20.6_ and IST_7.6_ in the parafoveal VD of affected sector was significantly associated (*P* = 0.014) with foveal thickening (Table 2, Figures 3 and 4).

**Table 2.** Association Between Differences in Parafoveal Vessel Densities Examined With IST of 20.6 msec and Those With IST of 7.6 msec and Retinal Thickness Changes in the Corresponding Parafovea and Fovea

	Corresponding Parafovea		Fovea	
	R	*P* *	β	*P* †
Affected	0.409	0.020	0.454	0.014
Unaffected	0.130	0.477	0.151	0.406
Nasal	0.442	0.011	0.079	0.776
Temporal	0.114	0.543	0.079	0.769

* Univariate linear regression analysis was performed to evaluate the contribution of the parafoveal vasculature to the corresponding parafovea thickness changes.

† A multivariate regression analysis was performed to evaluate the contribution of the parafoveal vasculature to the foveal thickness changes.

## Discussion

Using a VISTA protocol, we could detect the retinal blood flow changes in eyes with BRVO; these could not be imaged well using conventional OCTA alone. In addition, we identified that the parafoveal circulatory changes in the affected sector were associated with the retinal thickening in the corresponding parafovea and the fovea.

The relationship between macular nonperfusion and ME has been somewhat controversial in eyes with RVO.^22–24^ Yoo et al^22^ suggested that larger area of retinal nonperfusion might be a risk factor for ME recurrence. By contrast, Finkelstein^23^ showed that macular nonperfusion might contribute to the spontaneous resolution of ME and a better concomitant visual outcome. We suggest that the inconsistencies in the previous reports might have resulted from variations in the assessments of the retinal nonperfusion. On FA for eyes with RVO, the assessment of retinal nonperfusion (capillary dropout) is difficult because the affected retinal areas often experience dye leakage, which limits image resolution and contrast.

Retinal vascular changes can be evaluated more clearly and objectively using OCTA because it is not susceptible to dye leakage and it has a higher resolution. Optical coherence tomography angiography might be a more suitable than FA for the evaluation of the capillary changes associated with RVO. However, researchers have reported that microaneurysms that were detectable on FA could not always be delineated on OCTA. Fluorescein angiography visualizes retinal vasculatures based on the dye (plasma) dynamics; OCTA is based on erythrocyte dynamics. The difference in imaging mechanisms may relate to the detection rate of the retinal vascular changes; it is possible that the retinal blood flow is too slow to be visualized using an IST in the default setting.

Choi and Moult et al^18^ recently developed the VISTA technique for OCTA, which enables capture of the relative blood flow speed by shortening the IST from the default setting to 1.5 milliseconds and 3 milliseconds. Using their VISTA protocol, new findings were identified in diabetic retinopathy^19^ and age-related macular degeneration (polypoidal choroidal vasculopathy and geographic atrophy).^20,21^ By contrast, our VISTA protocol differed from that of previous reports and was a new attempt to delineate slowed blood flow by extending the IST from 7.6 milliseconds to 20.6 milliseconds. Our VISTA protocol facilitated detection of the slowed retinal blood flow in the affected sector (Figures 2 and 3 and see **Table**, **Supplemental Digital Content 1**, http://links.lww.com/IAE/B762); this was found to be significantly associated with future retinal thickening in the corresponding parafovea and fovea (Figures 3 and 4 and Table 2).

In this study, ME subsequently recurred in some patients whose parafoveal VD at IST_7.6_ in the affected sector was slightly low and the difference in the parafoveal VDs (IST_20.6_ – IST_7.6_) was large (Figure 3, upper panel). By contrast, ME did not recur in other patients whose parafoveal VD at IST_7.6_ was quite low and the difference in the parafoveal VDs (IST_20.6_ – IST_7.6_) was almost none (Figure 4, upper panel). Previous reports have shown that the larger macular nonperfusion area may contribute to the resolution of ME.^11,12,23^ In this study, some cases with low parafoveal VD at IST_7.6_ in the affected sector had large macular nonperfusion area in the corresponding parafovea. In such cases, the parafoveal VDs at IST_20.6_ did not increase, and the differences in the parafoveal VDs (IST_20.6_ − IST_7.6_) were faint. A lower parafoveal VD at IST_7.6_ does not always imply large differences in the parafoveal VDs (IST_20.6_ − IST_7.6_).

The retinal vasculature that was detectable using our VISTA protocol might cause tissue hypoxia and upregulation of VEGF, which could increase vascular permeability and retinal exudative changes. Using conventional OCTA (without VISTA) for eyes with BRVO, Kogo et al^13^ reported that the sectoral vessel dilation (increased vessel diameter index) at the parafovea was positively associated with ME, similar to the current VISTA finding. Using an adaptive optics scanning laser ophthalmoscope and a retinal function imager, the blood flow velocities of the capillaries and venules at the human macula have been found to be 1.34 to 1.49 mm/second,^25,26^ and 2.82 to 3.70 mm/second,^27^ respectively. By contrast, the lowered thresholds for blood flow velocity measurement at IST_7.6_ and IST_20.6_ in our VISTA protocol provided calculations of approximately 1.13 mm/second and 0.42 mm/second, respectively (4,000/464 = 8.62 um/pixel, vs. [mm/second] = 8.62/IST). Thus, the range of the blood flow velocities of the newly detectable vessels in our VISTA protocol (enabled by extending the IST from 7.6 milliseconds to 20.6 milliseconds) is estimated to be 0.42 to 1.13 mm/second. Combined with the results of previous reports, we suggest that the VISTA results of this study demonstrated that an increase in the parafoveal VD (IST_20.6_ − IST_7.6_) is a good representation of how congestive blood flow is affected by BRVO.

In the current study, the delineation of most abnormal vessels that morphologically appeared to be collateral vessels was hardly altered in the extension of IST. However, in a few collateral vessels, the parafoveal VD at IST_20.6_ was slightly greater than that at IST_7.6_, and their delineation was enhanced in the extension of the IST. In such vessels, the blood flow may be congestive. Further studies are needed to examine the association between the morphologic and functional changes in the collateral vessels and the recurrence of ME.

There are several limitations to the study that should be considered. The first and major limitation is that this was not a prospective study; the timing of the OCTA VISTA, duration after the onset, follow-up period, and the number of treatments varied among patients. Another possible limitation may be that the number of patients included was small, although some of the results were statistically significant. The third limitation is that the VISTA technique requires more imaging time compared with the conventional OCTA. It takes approximately 4 seconds to obtain OCTA images at IST_7.6_ and approximately 12 seconds at IST_20.6_. As extension of the IST may possibly be associated with a risk of poor fixation, which may result in poorer image quality, care should be taken to minimize the imaging time. However, the OCTA used in this study is equipped with a mode to automatically rescan the area that could not be captured in the first scan. Furthermore, speckle noise of the OCTA images could be sufficiently reduced by averaging three OCTA images. We considered that the image qualities of the OCTA obtained at IST_7.6_ were equivalent to those that were obtained at IST_20.6_. Despite these limitations, our VISTA protocol successfully detected slowed retinal blood flow at the parafovea, which was not detectable using the default settings. Moreover, the perfusion changes at the parafovea were found to contribute to the future development of the ME. The OCTA device used in this study is commercially available, and most of the results were analyzed automatically using the manufacturer's built-in software program, which is considered highly reliable.

Our VISTA protocol might also be applicable for examining the macular perfusion status in eyes with central RVO or retinal vascular diseases other than RVO. Further prospective studies with larger cohorts are necessary to confirm our VISTA findings.
